# Maternal-Fetal Interplay in Zika Virus Infection and Adverse Perinatal Outcomes

**DOI:** 10.3389/fimmu.2020.00175

**Published:** 2020-02-14

**Authors:** Franciane Mouradian Emidio Teixeira, Anna Julia Pietrobon, Luana de Mendonça Oliveira, Luanda Mara da Silva Oliveira, Maria Notomi Sato

**Affiliations:** ^1^Laboratory of Dermatology and Immunodeficiencies, LIM-56, Department of Dermatology, School of Medicine and Institute of Tropical Medicine of São Paulo, University of São Paulo, São Paulo, Brazil; ^2^Department of Immunology, Institute of Biomedical Sciences, University of São Paulo, São Paulo, Brazil

**Keywords:** maternal-fetal, neonatal, Zika virus, adverse effects, congenital infections

## Abstract

During pregnancy, the organization of complex tolerance mechanisms occurs to assure non-rejection of the semiallogeneic fetus. Pregnancy is a period of vulnerability to some viral infections, mainly during the first and second trimesters, that may cause congenital damage to the fetus. Recently, Zika virus (ZIKV) infection has gained great notoriety due to the occurrence of congenital ZIKV syndrome, characterized by fetal microcephaly, which results from the ability of ZIKV to infect placental cells and neural precursors in the fetus. Importantly, in addition to the congenital effects, studies have shown that perinatal ZIKV infection causes a number of disorders, including maculopapular rash, conjunctivitis, and arthralgia. In this paper, we contextualize the immunological aspects involved in the maternal-fetal interface and vulnerability to ZIKV infection, especially the alterations resulting in perinatal outcomes. This highlights the need to develop protective maternal vaccine strategies or interventions that are capable of preventing fetal or even neonatal infection.

## Introduction

Pregnancy represents a unique immunological condition with several regulatory mechanisms that ensure the non-rejection of the semiallogeneic fetus and its development, but it is also a time of greater vulnerability to infections (1). Associated with this, neonates also have a developing immune system with qualitative and quantitative differences from adults and poor immune memory, which increases their susceptibility to infectious agents (2, 3).

Complications during pregnancy, such as viral infections, can directly affect maternal-fetal health, since some viruses can be transmitted vertically and cause congenital infections (1, 4, 5). In addition, maternal immune activation induced by many common viruses is sufficient to cause neurological changes in the offspring (6).

In recent years, Zika virus (ZIKV) infection (Box 1) has been widely recognized because of its association with unprecedented cases of fetal microcephaly associated with congenital ZIKV syndrome (CZS) reported during the Brazilian epidemic in 2015 (5, 14). In the Americas, almost 80% of the ZIKV-associated microcephaly cases occurred in Brazil (15).

Box 1 **Key points of the Zika virus (ZIKV).**

ZIKV is a Flavivirus that includes other important human pathogens, such as dengue virus (DENV), West Nile virus (WNV), yellow fever (YFV), and Japanese encephalitis. Its genome contains a single open reading sequence that encodes three structural proteins (capsid [C], premembrane/membrane [prM/M], and envelope [E]) and seven non-structural proteins (NS1, NS2A, NS2B, NS3, NS4A, NS4B, and NS5) (7, 8). Due to genotypic differences, the African and Asian lineages have been determined (9). ZIKV is transmitted mainly by *Aedes* mosquitoes, highlighting the *Ae. Aegypti* and the *Ae. Albopictus* as potential vectors for worldwide expansion (10, 11). Other documented routes of transmission include sexual, intrauterine, perinatal exposure and breastfeeding (12, 13).

The main congenital infections are related to the TORCH group of pathogens, which include *Toxoplasma gondii*, others (*Listeria monocytogenes, Treponema pallidum*, human immunodeficiency virus, varicella zoster virus, enterovirus, and parvovirus), rubella, cytomegalovirus (CMV), and herpes simplex virus (HSV) (16). Due to its implication in fetal central nervous system abnormalities, the inclusion of the ZIKV to the TORCH acronym has been proposed (17).

Vertical transmission of ZIKV may occur during pregnancy (congenital transmission) or around the time of birth. Upon perinatal infection, the child may develop a maculopapular rash, conjunctivitis, arthralgia, and fever (18). While several mechanisms regarding congenital impairment and the pathogenesis of ZIKV infection in pregnant women have already been described, information on vertical transmission after delivery is still limited.

This article aims to review the immunological aspects of the maternal-fetal and neonatal interface that predispose susceptibility to infections, with an emphasis on the perinatal effects of ZIKV infection, and address maternal ZIKV vaccination studies, which offer a promising avenue.

## Immune Changes During Pregnancy

Pregnancy is a complex immune phenomenon that requires several adaptations of the maternal immune system to tolerate the semiallogeneic fetus. This tolerance is associated with mechanisms involving both a reduction in presentation of fetal antigens by placental cells and suppression of the maternal immune response. In this context, several factors act to favor the generation of regulatory responses and the attenuation of inflammatory and cellular activation (19). However, the maternal immune system needs to be finely balanced during pregnancy so that, while preventing fetal and placental rejection, it does not lose its ability to fight infections.

During pregnancy, hormonal variations occur that play an active role in the modulation of immune responses, leading to a reduction in the number of dendritic cells (DCs) and monocytes, a decrease in macrophage activity, and the inhibition of natural killer (NK), T, and B cells (20). Hormones and anti-inflammatory cytokines [transforming growth factor (TGF-) β and interleukin- (IL-)10] are highly produced during this period and may further increase the tolerogenic potential of maternal DCs by favoring the generation of Th2 profile responses (21, 22).

In addition to systemic changes, many immune response regulation mechanisms are present in the placenta that represent the maternal-fetal interface, where fetal antigens are in close contact with maternal blood. These sites are largely inhibited by maternal immune cells in the decidua, which is the endometrial layer that forms in preparation for pregnancy (23). In addition, fetal placental cells that form the villi also play important immunoregulatory roles (24).

### Decidual Immune Cells

The decidua is rich in leukocytes, especially uterine decidual NK (dNK) cells, macrophages, and T cells (23, 25).

dNK cells represent ~70% of the leukocytes present in the decidua during the first trimester of pregnancy and have a crucial role during placentation (23, 25), acting with trophoblastic cells (cytotrophoblasts, syncytiotrophoblast, and extravillous trophoblasts) during this process (26). The plasticity of dNK cells is crucial for successful pregnancy, and while these cells are important for orchestrating the invasion of trophoblast cells and spiral artery remodeling, they also protect the fetus from pathogens by destroying infected cells (26).

dNK cells originate from NK cell precursors, hematopoietic cells, or peripheral NK cells that migrate to the uterus during pregnancy and undergo phenotypic and functional modifications (27, 28). They are characterized by low cytotoxic capacity but high production of immunoregulatory cytokines (29), matrix metalloproteinases (30), and angiogenic factors (31) that contribute to tissue remodeling early in pregnancy. This limited cytotoxic activity may be attributed to defective immunological synapses (32) or to the expression of inhibitory variants of 2B4, NKp44/NCR2, and NKp30/NCR3 receptors (33, 34).

dNK cells have unique transcriptional profiles, in which multiple genes are expressed that are completely absent in peripheral NK cell subsets. Some genes encode proteins that are involved in maternal-fetal tolerance (29). In addition, dNK cells demonstrate differential and controlled roles of activating receptors that assure the outcome of pregnancy and in performing cytolytic functions upon viral infections (35, 36). This phenotype is even more pronounced in subsequent pregnancies and may be associated with improved placentation (37).

Decidual macrophages are also widely distributed in this niche, making up ~20–25% of local leukocytes in early pregnancy (38). These cells exhibit an immunoregulatory profile that favors angiogenesis and tissue remodeling and promotes the phagocytosis of apoptotic cells, preventing the release of proinflammatory mediators (39). These cells also prevent trophoblast lysis by dNK cells through TGF-β production (40).

DCs present in the decidua have an immature phenotype, with tolerogenic properties in both the first and third trimesters of pregnancy (41). Changes that lead to decreased immature DCs and increased mature DCs are associated with gestational pathologies (42). A peculiarity of decidual DCs is the low secretion of the Th1 proinflammatory cytokine IL-12 compared with that of circulating DCs, which favors prevalence of the Th2 response profile and fetal tolerance (43). DCs also stimulate dNK cell proliferation and activation (44), and their absence leads to impairment of the implantation process, angiogenesis, and decidual differentiation, as well as resorption of embryos (45). Together, these findings suggest that, despite being a rare population, decidual DCs are critical to gestational success.

Another population that is present in the decidua is T cells, which represent 10–20% of local leukocytes and include CD4^+^ and CD8^+^ T lymphocytes (25). Recently, it was verified that CD8^+^Tim-3^+^CTLA-4^+^ T cells found in the decidua play an immunosuppressive role through anti-inflammatory cytokine production, and a reduction in this population is associated with the occurrence of miscarriage (46). For CD4^+^ T cells, there is a prevalence of the Th2 profile response in the decidua due to the production of IL-13, IL-10, IL-4, and IL-6 by fetal placental cells, the amnion, and the decidua itself (21).

In addition, there is an increase in regulatory CD4^+^ T (T_reg_) cells, which is dependent on estradiol and exposure to paternal antigens (47, 48). These cells play a key role in maintaining tolerance by suppressing autoreactive lymphocytes and paternal antigens through TGF-β and IL-10 production. T_reg_ cells are also found in the decidua, attracted by the production of chemokines and chorionic gonadotropin (49, 50). In humans, decidual T_reg_ cells increase during the third trimester of healthy pregnancies, and their decrease is associated with the development of preeclampsia (51).

### Immune Cells in Placental Villi

In addition to maternal tolerance mechanisms, placental fetal cells have also developed strategies to prevent rejection during pregnancy and play an immunomodulatory role. Trophoblasts do not express major histocompatibility complex (MHC) class II molecules due to a failure in the expression of the CIITA regulator (52), and the expression of these molecules by this cell type is associated with villitis, recurrent miscarriage, and gestational pemphigoid (53–55).

Trophoblasts do not express classical MHC Ia molecules (A, B, and C) even under IFN-γ stimulation, which decreases recognition and destruction by maternal immune cells (56). However, extravillous trophoblasts, which invade the decidua, express MHC Ib molecules (E, F, and G) that have tolerogenic properties and modulate T and NK cells, macrophages, and DCs (57–59). These fetal cells may also release microvesicles that carry immunomodulatory proteins, such as fibronectin, syncytin, galectin-3, human leukocyte antigen G (HLA-G), and prostaglandins (60).

The placental villi have resident macrophages, namely, Hofbauer cells (HBCs), which have an M2 regulatory phenotype and play an essential role in controlling the inflammatory response in the course of pregnancy (61). HBCs expressing DC-SIGN produce IL-10 and are decreased in preeclampsia cases (62).

Due to the remarkable tolerogenic response, pregnancy becomes a period of greater susceptibility to infections that represent a risk not only to the mother but also especially to the fetus, who may develop more severe manifestations of various diseases (63). These infectious complications can promote a tolerance break and induce an inflammatory process. In an experimental model, it was found that stimulation with Toll-like receptor (TLR) agonists and consequent induction of IFN-γ and TNF induce miscarriage (64). Inflammatory activation during pregnancy is associated with risks of fetal abnormalities, such as ventriculomegaly and hemorrhage, as well as the development of neuronal diseases, such as autism, schizophrenia, and other conditions (5, 65–67).

In general, the placenta (and its immune cells) play a protective role in inhibiting the transmission of pathogens to the fetus by separation of maternal and fetal vascular supplies, the trophoblastic barrier and HBCs. However, the placenta may also play a permissive role in the transmission of infectious agents to the fetus, as occurs with ZIKV infection (68).

## Fetal and Neonatal Immunity

Infectious diseases and neonatal complications, such as prematurity and malnutrition, are responsible for most infant deaths worldwide, and it is estimated that 47% of deaths in children under 5 years old occur within the first 28 days of life (neonatal period) (69).

Fetal and neonatal immune responses are recognized by qualitative and quantitative differences from adult immune responses in almost all aspects of immunity. Therefore, susceptibility to early-life infections is associated with fetal and neonatal immunological immaturity, which, while contributing to maintaining tolerance during pregnancy, is not fully able to combat infections (70). However, in certain situations *in vitro*, neonatal cells are able to respond similarly to adult cells, highlighting the plasticity of the neonatal immune system (2, 3).

Many studies have shown the onset of immune cells during fetal development. In humans, fetal macrophages come from the yolk sac (71) and migrate to target tissues such as the central nervous system, resulting in resident macrophages (72). Tissue macrophages, such as alveolar macrophages and Langerhans cells, can also be derived from fetal liver monocytes (73, 74).

During human fetal development, T cells have been found in the thymus beginning at the tenth gestational week (75). Antigen presenting cells (APCs) play an important role in the profile of generated T lymphocytes, which are much more likely to generate T_reg_ cells. Additionally, maternal cells cross the placenta and settle in the fetal lymph nodes, stimulating the generation of T_reg_ cells, which contribute to fetal tolerance (76). Interestingly, ~6–7% of fetal thymocytes are T_reg_ cells (77), and these cells can still be found in high proportions (15–20% of CD4^+^ cells) in fetal secondary lymphoid organs during the second gestational trimester in humans (78). In addition, fetal DCs promote T_reg_ induction and inhibit T cell tumor necrosis factor- (TNF-) α production through arginase-2 activity (79). Moreover, fetal liver NK cells inhibit Th17 cells by IFN-γ production, helping to maintain a tolerogenic environment (80).

Even after birth, several peculiarities can be observed regarding neonatal immune cells. Monocytes, DCs, and macrophages secrete reduced TNF-α, IL-12p70, and IFN-α and express reduced CD80, CD86, and MHC II after activation via TLR (81–83). There is also a reduction in secretion of IL-18 by DCs, which acts together with IL-12 and IFN-type I to activate NK cells (84). However, the secretion of IL-1β, IL-6, IL-23, and IL-10 is similar or even higher than the level in adults (81), suggesting that neonatal DCs have the ability to secrete cytokines, but their response under stimulation differs from that of adults.

Neonatal immune cells are also characterized by lower IFN-I production under viral stimulation or TLR agonists (82), possibly due to a reduced interaction of transcription interferon regulatory factor 3 (IRF3) with the co-activator CREB and DNA (85). Reduced IFN-α and IFN-β production is also associated with diminished translocation of the transcription factor IRF7 into the nucleus in neonatal plasmacytoid DCs (86). Additionally, macrophages derived from cord blood monocytes show reduced IL-6 and TNF-α production when exposed to respiratory syncytial virus (87). These cells are hyporesponsive to IFN-γ activation due to decreased signal transducer and activator of transcription- (STAT-) 1 phosphorylation (88) and produce increased levels of IL-27 cytokine to regulate IDO expression, which promotes immune tolerance by suppressing T cell responses (89).

Several factors contribute to the immaturity of the neonatal adaptive response, such as the absence of an appropriate anatomical microenvironment for T and B cell interactions in lymphoid tissues, reduced ability of T cells to regulate CD40L expression, and low expression of adhesion molecule receptors (LFA-1, LFA-3, and CD2) and MHC molecules (3). Follicular DCs in neonates are also slow to form germinal center sites in secondary lymphoid organs and to promote B cell activation and proliferation (90). Such characteristics lead to a late production of T-dependent antibodies, which have lower affinity and shorter duration responses compared to those of adults (3).

Newborns have a reduced response to T-independent antigens, such as bacterial polysaccharides, possibly due to reduced TACI expression in B cells (91). Another peculiarity is the predisposition to develop predominantly Th2 responses to live or attenuated viral immunizations (92) due to decreased secretion of Th1 cytokines (IFN-γ and IL-12) and epigenetic configurations that favor IL-4 and IL-13 production (93, 94). In the fetal/neonatal period, there is more susceptibility to infections, especially to viruses that may cause neurological impairment.

Among the main fetal complications, infections by the TORCH group are the most common, and they are associated with 2–3% of congenital abnormality manifestations (95). These pathogens are present in the maternal bloodstream and can be transmitted hematogenously to the fetus through the placenta, resulting in an intense local inflammatory process (96).

Some of these infections can be prevented or controlled, such as congenital rubella infection, which has ceased to occur in countries with immunization against the virus (97), or maternal syphilis infection, which can be easily treated during pregnancy when discovered early, diminishing the deleterious effects on the fetus (95).

In this context, congenital HSV infection has an important impact because it can be disseminated and is associated with a high degree of mortality (98). In addition to skin and mouth rashes, fever, and eye problems, neonates can develop central nervous system infections causing encephalitis in the first month of life (98).

CMV infection until the second trimester of pregnancy also causes neuronal damage to the fetus (99). Among the main neuronal symptoms, we can highlight sensorineural hearing loss in children and neurodevelopmental delay, in addition to microcephaly; however, 85–90% of infected newborns present the asymptomatic form of the disease (100, 101). Congenital rubella infection can also cause hearing loss in addition to neurological changes, such as meningoencephalitis and microcephaly (102), when it occurs during the first trimester of pregnancy. However, other infections do not have an effective treatment or diagnosis and, despite presenting mild manifestations in the pregnant woman, can generate fatal manifestations in the fetus.

Neonatal infection occurs mainly during childbirth (85%) but can occur during the intrauterine (5%) and postnatal (10%) periods (98). Perinatal infections can occur through vertical transmission from a viremic mother to her newborn during pregnancy, delivery, breastfeeding, and close contact between the mother and her baby, as evidenced for ZIKV (103).

Perinatal transmission has already been reported for other arboviruses, such as DENV (104, 105), WNV (106), Chikungunya virus (CHIKV) (107), and YFV (108). The evidence for perinatal transmission of ZIKV was described in 2013 (109), prior to the recent epidemiological outbreak in Brazil.

The contemporary strain of ZIKV has a single serine to asparagine substitution (S139N) in its prM polyprotein that makes it more neurotropic because of an increase in its infectivity to neuronal progenitors and consequent microcephaly, which was confirmed in mice (110). It is also worth mentioning that this mutation emerged before the 2013 outbreak in French Polynesia.

Maternal ZIKV infection during pregnancy is usually asymptomatic for the mothers and can induce severe damage to the fetus similar to that found in the TORCH infections, such as fetal microcephaly, which was reported during the Brazilian epidemic in 2015 (111). Additional damage may include eye injuries, fetal growth restriction, and congenital contractures (112).

Altogether, this makes it vital to develop research for a better understanding of the pathology of ZIKV infection and to design vaccines and treatments that are appropriate for pregnancy.

## Brief Overview of ZIKV Infection

ZIKV infection is an emergent aggravating factor of public health issue worldwide, representing a high morbidity rate that is mainly associated with congenital infection. Most cases of ZIKV infection occur by the vector route through the bite of the *Aedes* mosquito (Box 1). However, confirmed cases of non-vector infection, including sexual transmission, blood transfusion, and vertical transmission, have been reported (12). Sexual transmission has been confirmed in more than 13 countries in which sex partners have coincidentally traveled to epidemic regions, and in most cases, transmission occurs from male to female (113).

In this context, in ZIKV-infected women, viral particles disappear from the vaginal tract 3 weeks after symptoms, although the virus persists in the bloodstream (114). In men, it was verified that ZIKV persists in the semen for ~120 days and for 34 days in urine, whereas only 5% of patients had the virus in their saliva (115). Another study showed that virus persists for up to 100 days in blood and 168 days in semen, and it was associated with major production of soluble factors, including proinflammatory cytokines at early stages of infection (116). ZIKV infection has also been shown to modify semen characteristics, with late sperm motility and the presence of the replication-competent virus (117). This fact reflects public health implications because it may contribute to sexual transmission, especially in couples who wish to have children (117).

Male genital tract cells are highly permissive to the ZIKV; this fact still remains underexplored but could lead to male infertility, as a “break” in the immune privilege of the testes could compromise spermatogenesis (118). Although the long-term effect on male fertility remains unclear, men who were tested 12 months after the symptoms of infection, were negative for ZIKV by RT-PCR, but the sperm count was abnormal in 80% of the cases, including low sperm concentration and impaired motility (119).

The possibility of sexual transmission of ZIKV made headlines following the recent outbreak in the Americas, where most cases occurred in people who had symptoms but were asymptomatic at the time of intercourse (120). The growing number of cases of sexual transmission led the Centers for Disease Control (CDC) to recommend that people who had traveled to endemic areas abstain or have sex using a condom for at least 8 weeks for women and 6 months for men to prevent sexual transmission (121).

In adults, the infection is usually asymptomatic, and the symptoms are non-specific and last between 2 and 7 days, which makes diagnosis difficult. The main symptoms include fever, headache, vomiting, lymphadenopathy, a maculopapular rash, and conjunctivitis (122). One of the most alarming consequences of infection is the ability of the virus to attack mature or developing neuronal cells (123).

In rare cases, it can cause Guillain-Barre Syndrome (GBS), which is characterized by an acute inflammatory polyradiculoneuropathy resulting in weakness in the limbs and cranial nerves, with consequent physical limitations (124). People with GBS usually have more significant weakness within 2–4 weeks after symptoms begin, and as the disease progresses, the weakness may develop into paralysis. In some cases, the disease may result in neuromuscular respiratory failure (125).

Although ZIKV infection is asymptomatic or mild in most cases, infection during pregnancy can cause fetal developmental defects, such as microcephaly and other severe brain abnormalities.

## Infection in Pregnant Women and Congenital Effects

ZIKV infection causes microcephaly and other neurological malformations that characterize CZS. In the beginning of the epidemic in 2015, ZIKV triggered a microcephaly outbreak in northeastern Brazil, where the incidence was 20 times higher than during other periods, and viral RNA was found in the fetal brain tissue of newborns with microcephaly, as well as in the amniotic fluid and placenta of mothers (126). It was estimated that 10% of babies of infected mothers had some birth defect (127).

At the peak of the epidemic in 2016, 216,207 probable cases of acute ZIKV disease were reported in Brazil, and it is estimated that 8,604 babies were born with malformations (128). In 2019, 1649 probable cases of ZIKV infections were reported in pregnant women, 447 of which were confirmed by laboratory tests (129).

Microcephaly is defined by the World Health Organization (WHO) as a reduction in head circumference (occipitofrontal diameter) in infants born at 37 or more weeks of gestation, with a measurement for boys equal to or <31.9 cm and for girls equal to or <31.5 cm (130). CZS is characterized by severe microcephaly and others neurological lesions, ocular findings, and congenital contractures. Additionally, other abnormalities described include craniosynostosis, fetal growth restriction, craniofacial malformations, pulmonary hypoplasia, and arthrogryposis (112).

For other arboviruses, severe consequences of maternal-fetal transmission have been reported, notably for CHIKV (encephalopathy and hemorrhagic fever) (107) and DENV (preterm birth, fetal death, low birthweight, and fetal abnormalities) (131). In a murine experimental model, it was reported that intrauterine ZIKV infection led to placental dysfunction and perinatal effects (132).

**Figure 1:**
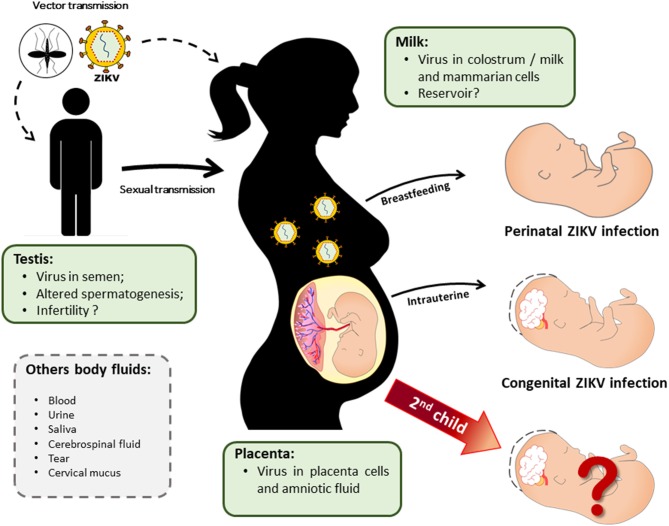
ZIKV vertical transmission and a possible maternal reservoir. ZIKV infection in pregnant women may occur by mosquito bite or sexual contact with an infected partner. Mother-to-child transmission can either occur *in utero* (infection in the first trimester of pregnancy is related to congenital ZIKV syndrome [CZS]) or in the perinatal period via breastfeeding. ZIKV presents tropism for multiple tissues and is present in several body fluids, which contribute to its transmission by different routes. However, after gestational infection with the congenital involvement of the child, it is still unknown whether the ZIKV establishes a reservoir in the mother that may influence the course of a second pregnancy.

Manifestations due to mother-to-child transmission can be detected early, denoting the clinical and laboratory aspects of anomalies prior to birth, at the moment of delivery, or later, indicating that diseases may appear months or even years after the birth of congenitally infected babies (102) (Figure 1).

In CZS, most of the abnormalities reported in the first cases were calcifications at the cortical–subcortical junction of the white matter and malformations in cortical development associated with other abnormalities (133, 134). Other authors have described changes such as parenchymal atrophy (and secondary ventriculomegaly), subependymal pseudocysts, agenesis/hypoplasia of the corpus callosum, parenchymal calcification, cerebellar and brainstem hypoplasia, lissencephaly–pachygyria, and cortical laminar necrosis (112, 135).

Eye injuries were also detected in CZS newborns; most cases occurred in babies with a small cephalic diameter at birth and in mothers who reported symptoms in the first trimester of pregnancy (136). In different regions where the viral outbreaks occurred, similar lesions were found, such as macular lesions, optical nerve abnormalities, chorioretinal atrophy/scarring, focal pigment mottling of retina, microphthalmia, glaucoma, cataract, iris coloboma, and subluxation (112, 137).

In a prospective cohort study involving 216 infants, 31.5% of children between 7 and 32 months of age had below-average neurodevelopment (cognitive, language, and motor domain) and/or abnormal ocular or auditory assessments. There was also resolution of the microcephaly with normal neurodevelopment in two of eight children, the development of secondary microcephaly (children born with normal head circumference that develop microcephaly in the first year of life) in two other children, and autism spectrum disorder in three previously healthy children in the second year of life (138).

Other parameters were also evaluated, such as the eruption of primary teeth in 74 children with CZS for a period of 2 years, where 94.4% simultaneously exhibited two or more related signs and symptoms. Increased salivation, irritability, and gingival itching were the most commonly reported signs and symptoms (139).

A prospective study in progress aims to evaluate ~10,000 pregnant women from six endemic countries from the beginning of pregnancy up to 6 weeks after delivery and their children until they are 1 year old (140). This study will contribute to understanding the critical questions about the impact of intrauterine exposure to ZIKV, even without congenital abnormalities.

Finding a correlation between ophthalmological and neurological findings, as well as the attempt to standardize the syndrome and its correlation with other congenital infections, has become difficult due to the lack of a more accurate description of neuroimaging and ophthalmic results. Furthermore, the prospective study of children who have been exposed to ZIKV to assess other later effects has become indispensable.

### Mechanisms Involved in Congenital Infection

It is well-established that ZIKV infection during pregnancy can cause harm to the fetus (95). However, the mechanisms involved in congenital infection are still being studied in cohorts of children with or without microcephaly. Some data suggest that neonatal infection may be persistent and may occur at different stages of pregnancy.

More severe damage to fetal development, such as microcephaly, has been shown when maternal ZIKV infection occurs in the first trimester of pregnancy (141). Histopathological findings in the placenta at this time were similar to those found in TORCH infections, including chronic villitis, edema, trophoblastic lesion, and an increase in HBCs (142). However, third semester placentas showed only delayed villous maturation without any pathological changes. In addition, ZIKV RNA was also detected in the placentas of infected patients in the second and third trimesters (142). Studies in mice have also shown greater congenital damage when infection occurs in the early stages of pregnancy (143, 144).

Additionally, inflammatory and necrotic lesions of the placenta that are typical of other TORCH infections do not occur in placentas from the second and third trimesters with intrauterine ZIKV infection (96). On the other hand, these placentas do present proliferation and hyperplasia of HBCs.

Regarding the transplacental passage of ZIKV, some studies suggest that the autophagy pathway participates in the formation of microvesicles carrying the virus (145). In a primary culture of murine neurons, an increase in exosome production was observed following ZIKV infection through the expression of neutral sphingomyelinase (nSMase)-2 or SMPD3, which regulate the production and release of exosomes (146). SMPD3 silencing reduced the viral load, showing the important role for exosomes in ZIKV infection.

Another vertical transmission mechanism is placental translocation of infected cells or free virions (147). ZIKV is capable of infecting several primary cells and explants of the human placenta (cytotrophoblasts, endothelial cells, fibroblasts, HBCs, amniotic epithelial cells, and trophoblast progenitors), which have high expression of AXL, Tyro3, and TIM1—input receptors of ZIKV—in their membranes. These cells, especially HBCs, migrate to the placental–fetal interface and infect the fetus (148). In addition, the use of Duramycin, a peptide capable of binding to enveloped virions, prevents virion binding to the TIM1 receptor, resulting in reduced ZIKV infection in placental cells and explants. In addition, ZIKV has a greater tropism to the placenta and the most prominent cytopathic effects during the first trimester of pregnancy (68).

However, trophoblasts isolated from term placentas were found to be resistant to infection due to the constitutive production of type III IFN (IFN-λ1), which also ensured the protection of other cells because IFN-λ1 prevents infection in paracrine and autocrine manners (149).

In humans, the ZIKV NS5 protein antagonizes IFN signaling by binding to STAT2 and promoting its proteosomal degradation (150). STAT2 signaling appears to be relevant in the response against ZIKV, as STAT2-deficient mice are also susceptible to infections (151), as well as mice that are deficient in type I IFN receptors.

Once infected cells or virions come into contact with the fetal interface, ZIKV infects fetal cells and may cause teratogenic effects, such as microcephaly. Viral RNA was detected in the placentas of 9 out of 12 ZIKV-infected mothers who had miscarriages at up to 19 weeks of gestation (152). ZIKV RNA was also detected in the brain of 7 out of 8 fetuses with microcephaly, showing that early pregnancy infection may favor a replicative niche of the virus in the placenta, contributing to teratogenic effects.

ZIKV has the potential to cross the blood–brain barrier, as evidenced *in vitro* (153), due to AXL expression in endothelial cells. In addition, ZIKV has tropism to neuronal progenitor cells (154), astrocytes, oligodendrocytes, mature neurons, reproductive organ tissue cells (uterus, vagina, and testis), eye tissue (ganglion cells, optic nerve, and cornea), fluids (tears, saliva, semen, and urine), hepatocytes, epithelial cells, fibroblasts, renal cells, peripheral blood mononuclear cells, and neutrophils (155).

ZIKV tropism to neuronal progenitor cells leads to the manifestations observed in CZS (microcephaly, cerebellar malformation, brainstem, thalamus, and ocular impairment) (156), which are related to the cellular damage caused by ZIKV infection. Following *in vitro* ZIKV infection, cortical neuronal progenitors show unregulated cell replication cycles, decreased cell division rate, and increased apoptosis (157). ZIKV is capable of breaking double-stranded DNA and inhibiting DNA repair pathways during the cell replication cycle (158). In addition, permanence in phase S increases viral replication in neuronal progenitors, favoring increased neuronal damage in CZS.

Another mechanism associated with neuronal progenitor cell death is TLR3 activation by ZIKV, which promotes dysregulation of genes involved in neurogenesis and apoptotic pathways (159). Curiously, the remaining infected neuronal cells appear to behave as reservoirs for the virus without significant immunological activation, suggesting that there may be repercussions even several months after infection (160).

It is noteworthy that although ZIKV is able to infect a wide variety of cell types, and some cells are more permissive than others. In this context, ZIKV most effectively infects the most differentiated neuronal progenitor cells (161) and modulates Notch pathway gene expression, which is involved in cell proliferation, apoptosis, and differentiation during neurogenesis.

Moreover, cases of dizygotic twin pregnancies in which only one of the neonates had CZS show that intrinsic factors, such as oligogenic and epigenetic mechanisms, also influence infection, since serodiscordance was not observed in monozygotic twins (162).

## Postnatal ZIKV Infection

The neonatal phase is characterized by a high susceptibility of neonates to infections due to their developing immune systems (163). During this period, neonates depend on maternal immunity via passive antibody transfer through breastfeeding. However, breast milk can be a potent vehicle for viral transmission, as has already been shown for flaviviruses such as DENV (164, 165) and WNV (165). Postnatal infection in children through breastfeeding has also been observed in ZIKV infection (Figure 1) (13, 166, 167).

ZIKV has been detected in breast milk and the mammary tissue of mice but there has not been transmission to the offspring via this route (168). Human case reports have confirmed the presence of ZIKV in breast milk and its transmission to newborns (13, 167). In addition, the presence of ZIKV was also evidenced in breast milk, as well as in the serum and urine of a newborn with congenital defects whose mother was infected early during pregnancy (169).

In one of the reported cases, an infected Brazilian woman at the end of pregnancy presented persistent virus in breast milk, with a high viral load and cytopathic effects, suggesting a high infectious capacity to her newborn (170). Breastfeeding was suspended, transmission to the child was not evidenced, and no abnormalities were associated with fetal development.

The persistence of the ZIKV viral load was also reported in cerebrospinal fluid and serum at 6 and 17 months of age in a case of severe microcephaly (171). However, the viral presence in breast milk was not evaluated. In addition, the clinical development of the infant consisted mainly of recurrent episodes of seizures, delayed neuropsychomotor development, dysphagia, visual impairment, and double spastic hemiplegia.

Although knowledge about the pathogenesis of ZIKV infection has expanded after the epidemiological outbreak in pregnant women in the Americas, there is still little information available on the clinical manifestations of ZIKV infection in the pediatric population.

A cohort study of 351 children under 18 years of age with confirmed ZIKV infection showed that in addition to fever, other frequent symptoms were rash (79.8%), facial or neck erythema (69.2%), fatigue (66.7%), headache (63.5%), chills (60.4%), pruritus (58.7%), and conjunctival hyperemia (58.1%) (172).

Most case studies of symptomatic pediatric ZIKV infection have shown a typically mild disease (172–174), similar to that observed in adult infection. However, complications associated with infection in newborns have rarely been reported (18), as well as ZIKV-related Guillain-Barré syndrome in adolescents (175).

**Figure 2:**
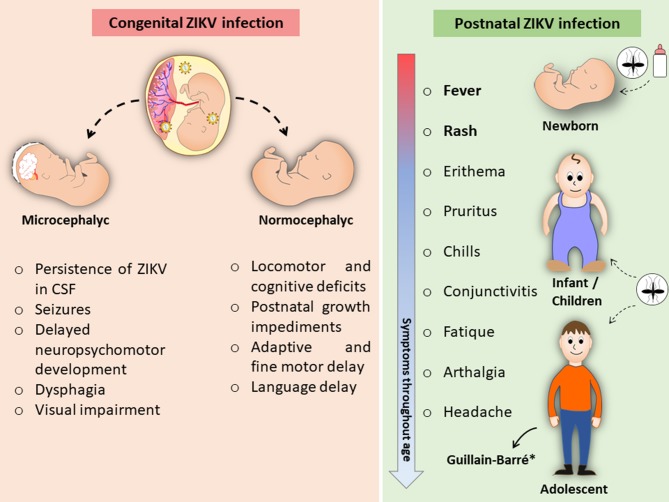
Adverse effects of congenital and postnatal ZIKV infections. Intrauterine exposure to ZIKV may lead to congenital infection causing fetal microcephaly, among other CZS-related effects. Even in infants who have not had microcephaly, congenital ZIKV infection can cause delays in locomotor and cognitive development. Pediatric ZIKV infection is self-limiting and typically causes mild and even asymptomatic disease similar to adult infection. ^*^Rare cases of ZIKV-associated Guillain-Barré.

Despite the impact of CZS, the consequences of congenital ZIKV infection in non-microcephalic newborns have received considerable attention (Figure 2). Among the babies that appear healthy at birth, there are increasing reports of postnatal developmental defects (176). In the United States, it has been described that 9% of 1-year-old babies from ZIKV-infected mothers had at least one neurodevelopmental abnormality, possibly associated with CZS infection (177).

In immunocompetent mice, mild congenital ZIKV infection in pups did not display apparent defects at birth, whereas manifestations of postnatal growth impediments and neurobehavioral deficits (locomotor and cognitive) persisted to adulthood (178). Postnatal effects of infection appear to be associated with the gestational period during which the infection occurred (179).

Prospective studies related to congenital ZIKV infection, as well as the long-term effects in children with or without CZS, are still under development. The few existing data show that microcephalic infants have important overall developmental delays, while in normocephalic infants other developmental complications have been observed, although neuroimaging findings have been normal (176). These complications include adaptive, fine motor, language, and personal–social delays.

## Maternal Immunization as a Protection Tool

Despite the reduction in the number of new ZIKV infections, the development of a safe protective vaccine is a public health priority. In the face of the recent ZIKV infection epidemic, several groups have developed different vaccine strategies (Box 2) that are capable of inducing high levels of neutralizing antibodies (nAbs), as well as generating protection against the challenge of infection in non-pregnant mouse and non-human primate models (184–186). However, in these studies, it has not been demonstrated whether this protection prevents congenital changes or has a long-term protective effect. Although several vaccine strategies have progressed into Phase I clinical trials in humans (187), gestational protection vaccine studies are still limited.

Box 2 **ZIKV vaccine approaches.**

Candidate vaccines include those based on inactivated or live-attenuated virus, viral vectors, DNA or RNA, virus-like particles, subunit vaccines, and viral proteins (180). Viral surface or envelope proteins are the most antigenic and often considered as the best candidates for immunization (181). In addition, E protein, composed of three domains (ED I/II/III), is the major inducer of neutralizing antibodies (nAbs) against ZIKV (182, 183).

Great progress has been made in the development of a ZIKV vaccine since the outbreak in 2015. Studies show that over 45 ZIKV candidate vaccines are in preclinical development (188). Currently, according to the WHO, 15 vaccine candidates are in Phase 1 clinical trials, two of which have now progressed to Phase 2 (189). Both of the Phase 2 vaccines are directed at the ZIKV prME immunogen. One is mRNA-based and indicated for adults and is sponsored by Moderna Therapeutics, while the other corresponds to a DNA model developed by National Institute of Allergy and Infectious Diseases (NIAID) and is for adults and children.

In neonates, active immunization tends to be less effective due to the immaturity of the adaptive immune system (3, 190). At this stage, children mainly depend on passive immunity by maternal antibody transfer. Thus, active maternal immunizations are a way to provide infants with passive immunity to specific diseases, with the potential to protect the mother, her fetus, and the infant during vulnerable periods in their lives (191).

Due to the immunological conditions present during pregnancy, vaccination in this period has evoked discussion. However, in recent decades, this approach has been increasingly used (191) and even proposed in ZIKV vaccination clinical trials (192). These vaccine strategies aim to induce a strong neutralizing humoral response that is able to prevent offspring from developing congenital and perinatal infections. Some candidate vaccines targeting females before ZIKV infection during pregnancy or in their offspring have shown satisfactory results (Table 1).

**Table 1:** Maternal vaccine strategies for ZIKV infection.

**Vaccine model**	**Immunization approach**	**ZIKV challenge**	**Key points**	**References**
mRNA (prM/E) and live-attenuated virus • Asian strain	Female wild-type C57BL/6 or A129 mice immunized before pregnancy	Embryo day 6 • African strain	Prevents placental damage and fetal demise	(193)
DNA (prM/E) • Asian strain	Female BALB/c mice immunized before pregnancy	Suckling mice • Asian strain	Inhibits the growth delay	(194)
Recombinant protein (ED III domain) • Asian strain	Female BALB/c mice immunized before pregnancy	Suckling mice • American strain	Protects from lethal challenge	(195)
Subunit (E) • Asian strain	BALB/c pregnant mice	Embryo day 13.5Suckling mice	Prevents microcephaly	(196)
Adenovirus vector-based (M/E) • Asian strain	Female A129 or Balb/c mice immunized before pregnancy	Embryo day 5.5 Pups • American strain	Prevents placental infection and fetal growth restriction	(197)

One of the first studies in this area used two different vaccine strategies and showed that maternal immunization of mice protected the fetus from vertical transmission of ZIKV when challenged during pregnancy, with the induction of high levels of nAbs that prevented placental damage and fetal demise (193). In addition, the vaccination of pregnant mice prevented the development of microcephaly in their offspring and generated a long-term protective response. This was evidenced in both *in utero* and neonatal challenges, with a reduction in ZIKV-infected cells in the brain (196).

In another model, maternal immunization of mice also prevented postnatal ZIKV infection and inhibited the growth delay of their offspring by increasing nAbs titers in the colostrum and offspring serum (194). It has also been reported that the immunization of female mice before pregnancy protected offspring from lethal challenge with two epidemic human ZIKV strains (195).

Recently, an adenovirus vector-based ZIKV vaccine provided robust maternal-fetal and postnatal protection against fetal ZIKV transmission *in utero* as well as in infants against ZIKV infection after birth (197).

In addition to prophylactic vaccine studies, the development of other therapeutic interventions is also necessary, especially for use in infected pregnant women. In this area, some groups have a neutralizing antibody therapy approach.

In mice, a study demonstrated that the passive transfer of serum from immunized females to offspring born to naive females and immunocompromised adults was able to protect against lethal ZIKV infection due to the cross-neutralizing activity of the serum (195).

Human monoclonal antibody studies have shown therapeutic efficacy in experimental models. A highly effective nAbs from a ZIKV-infected patient that was evaluated in pregnant and non-pregnant mice was able to prevent maternal-fetal transmission (182). Similarly, prior administration of a cocktail containing three potent ZIKV-neutralizing monoclonal antibodies to rhesus monkeys on the eve of the ZIKV challenge also completely inhibited viremia (198).

The current scenario highlights the importance of offering protection to women of childbearing age and their children by developing prophylactic maternal vaccines or interventions that are able to prevent pregnant women from developing ZIKV infection and, consequently, to prevent fetal/perinatal transmission.

## Conclusions and Future Perspectives

Prospective studies of children born to ZIKV-infected women are important for understanding the long-term effects of the disease. Follow-ups of children who are victims of intrauterine exposure will be critical in assessing the effects that may be influenced by the subtle immune responses triggered by ZIKV and in providing support for the early interventions that will improve the neurodevelopment of the children. In parallel, future studies of vaccine strategies that generate an optimal neutralizing antibody response are essential, considering that passive mother-to-child immunity against ZIKV during pregnancy could prevent congenital infection.

## Author Contributions

For the preparation of this review, FT, AP, LMO, LMSO and MS wrote, read and approved the final manuscript. FT drew the figures. FT and MS created the study concept and edited the manuscript.

### Conflict of Interest

The authors declare that the research was conducted in the absence of any commercial or financial relationships that could be construed as a potential conflict of interest.
